# The Structure of Children’s Subjective Well-being

**DOI:** 10.3389/fpsyg.2021.650691

**Published:** 2021-06-11

**Authors:** Shazly Savahl, Ferran Casas, Sabirah Adams

**Affiliations:** ^1^Centre for Interdisciplinary Studies of Children, Families and Society, University of The Western Cape, Cape Town, South Africa; ^2^Research Institute on Quality of Life, University of Girona, Girona, Spain; ^3^Faculty of Education and Social Sciences, Andres Bello University, Santiago, Chile; ^4^Centre for Higher Education Development, University of Cape Town, Cape Town, South Africa

**Keywords:** structure of children’s subjective well-being, children’s subjective well-being, Children’s Worlds Survey, hierarchical structural model, confirmatory factor analysis

## Abstract

Research on children’s quality of life and subjective well-being has advanced over the past decade largely as a result of developments in childhood theory, children’s rights legislation, and the shift toward positive social science. However, in line with the uncertainty regarding the conceptualization of subjective well-being, the structural configuration of children’s subjective well-being has not been considered in the literature. In the current study, we present and test a model of children’s subjective well-being, which includes global (context-free items assessing overall and general well-being, without reference to a specific aspect of life) and specific (domain-based items assessing a specific aspect of life) cognitive components, and positive and negative affect. We further test the fit structure of a hierarchical structural (second-order) model of children’s subjective well-being. Finally, we test the measurement invariance of the hierarchical model across age and gender. We use data from the third Wave of the Children’s Worlds Survey. The data source includes a sample of 92,782 participants selected from 35 countries (girls = 49.7%) in two age groups (10- and 12-years-old). We found a good fit for the four-factor confirmatory factor model of children’s subjective well-being. Correlations between the various latent factors were as anticipated—with positive correlations between the life satisfaction components and positive affect, and negative correlations with negative affect. We further found a good fit for the hierarchical structural model of children’s subjective well-being. Finally, we found the tenability of measurement invariance across age and gender. The study extends the generalizability of the hierarchical structural configuration of the subjective well-being to child samples, and provides a viable model to explore correlates and predictors of children’s subjective well-being using the full conceptual model. Finally, we propound the tenability of a quadripartite hierarchical conceptual model of children’s subjective well-being.

## Introduction

The concept of subjective well-being (SWB) has its genesis in the Greek philosophical concepts of “hedonia.” Hedonic well-being focuses on life satisfaction, happiness, and SWB; it is often denoted as “feeling well,” representing the good life, and concretized as experiencing happiness (Huta and Ryan, 2010; Adler and Seligman, 2016). The hedonic constructs of happiness and SWB present with substantial conceptual overlap and are frequently used interchangeably in the literature (Camfield and Skevington, 2008; Medvedev and Landhuis, 2018).

SWB is a multifaceted expansive concept, which consists of the cognitive and affective perceptions, experiences, reflections, and appraisals that people ascribe toward their lives (Diener, 1984, 2001). The cognitive component refers to global and domain-based life satisfaction, while the affective component refers to positive (PA) and negative affect (NA) (Diener et al., 1999). These components are conceptualized on a “tripartite” hierarchical structure that are conceptually aligned and moderately correlated (Diener, 2009). Casas (2017) theorizes that this model represents the first level of deconstruction if regressed onto the single item on life satisfaction: “How satisfied are you with your life as a whole”? (Cummins, 2000). However, regardless of the general acceptance of the components of SWB, there are still questions regarding the structural configuration—that is, how the various components of the construct fit together. Busseri and Sadava (2011) identified five structural configurations: as a separate components model, a hierarchical structure, a causal system, a composite model, and as a configuration of components. These various configurations of SWB have substantial theoretical, conceptual, and methodological implications and can lead to different conclusions concerning the relatedness and independence of the components, the predictors of SWB, and its stability of SWB over time. In an attempt at a resolution, Busseri (2015) examined these multiple structural configurations using cross-sectional and longitudinal data. They found that a hierarchical factor structure presented a viable structural conceptualization, fully accounted for the joint relatedness/independence of all three components of SWB, accommodated for the difference in this relatedness/independence observed in the cross-sectional vs. longitudinal findings, and completely addressed these issues when considering the predictors of SWB. Similarly, results supporting the stability of the hierarchical configuration was found in longitudinal and experimental studies (see Metler and Busseri, 2017), with both the shared and unique variances of the various components significantly contributing to the construct of SWB. Galinha and Pais-Ribeiro (2009) also found a good fit for a hierarchical model of SWB, which included global (context-free) and specific (domain-based) levels of measurement of cognitive SWB and state level positive and negative affect. They further confirmed the stability of the SWB structure across a 2-month replication period. A further contribution to the literature is made by Jovanovich (2015) who confirmed the tenability of a bifactor model with a general factor of SWB and three components of context-free cognitive life satisfaction, positive affect, and negative affect. More recently, Busseri’s (2018) meta-analytic review provided strong support for the generalizability and robustness of a hierarchical structural conceptualization of SWB. While these debates are well-grounded as it relates to SWB in adults, there is less consideration in the literature on SWB in children.

Research on children’s SWB has a much more recent history, which has advanced over the past decade largely as a result of developments in childhood theory, children’s rights legislation, and the shift toward positive social science (Savahl et al., 2015). These have been aggrandized by the *Child Indicator Movement* (Ben-Arieh, 2008) and further ameliorated by large-scale cross-cultural child well-being studies such as the Children’s Worlds survey (see Casas and Rees, 2015; Rees et al., 2020), the Multinational Qualitative Study on Children’s Understandings of Well-Being (Fattore et al., 2018) and research conducted by the Children’s Society (see Rees et al., 2012) and the Research Institute on Quality of Life at the University of Girona (see Casas, 2011). These studies provided the empirical basis that advanced, measurement, conceptual, and theoretical understandings of children’s SWB. Early research on children’s SWB used scales and measures adopted from adult versions. However, the field has seen rapid advancements; with recent studies (see e.g., Casas, 2017) demonstrating sound psychometric properties on a range of SWB scales used with children between the ages of 8- to 12-years. These advancements include locating the children centrally in the research process largely through innovative qualitative approaches (Fattore et al., 2018) and developing and adapting scales and measures that reflect direct engagement with children (see Casas et al., 2012; Montserrat et al., 2021). This growing body of scientific studies provide evidence of children’s cognitive capacity to meaningfully reflect on their lives in general and specific aspects of their lives, and to consider and endorse questions relating to their SWB. Indeed, Bradshaw (2019) goes as far as to suggest that recently more work has been done on developing multi-item scales on SWB for children than for adults. To date the research on children’s SWB, garnered from large-scale cross-cultural studies, have produced the following general findings:

1. Mean scores on children’s SWB are generally above the median (Klocke et al., 2014).
2. Mean scores on children’s SWB varies across countries (cultures) and are generally not comparable (Casas, 2017).
3. Child and adult SWB are relatively independent of each other and the factors that influence adult SWB and children’s SWB are distinct (Rees, 2017).
4. Much of the variations in children’s SWB remain unexplained (Bradshaw, 2019).
5. In-country comparisons of children’s SWB are more significant than between-country comparisons (Rees, 2017).

Comparisons across micro-level factors such as age and gender present with interesting trends. Scores on overall and domain-based measures of SWB tend to decrease with age; however, the mechanism driving this process is not clearly defined (see Casas and González-Carrasco, 2019 for a review); the tendency is more pronounced in girls (González-Carrasco et al., 2017). Across gender, the trend is less prominent. While girls tend to present with lower SWB scores than boys on overall SWB measures, there seems to be a differential experience across various domains of well-being and aspects of life (Rees, 2017; Rees et al., 2020).

Research on children’s SWB have tended toward a focus on the cognitive component, with measures of life satisfaction often used as a proxy for the overall concept of SWB. The evidence that the cognitive component is more stable than the affective component over time (Rees, 2018) likely drives this tendency. The measurement of SWB has also been a point of contention, with many large-scale studies relying on the use of single-item scales. For example, the Health Behavior in School-aged Children (HBSC) study and the Programme for International Students’ Assessment (PISA), both include single-item questions on Overall Life Satisfaction (OLS). Casas (2017), however, has questioned this practice, arguing from a psychometric perspective that multiple-item scales are more robust than single-item scales, especially when measuring non-observable constructs. Regardless, given its level of abstraction, researchers recommend the inclusion of the single-item scale on life satisfaction as a measure to assess convergent validity (International Well-Being Group, 2013). Others such as Davern et al. (2007) have argued that the best way to assess SWB is with the most abstract measures; while an alternative point of view reminds us that it may not be appropriate for use with younger children. On the issue of item abstraction, Casas (2017) found that domain-based SWB scales with items of varying degrees of abstraction, could potentially lead to improved model fit. Studies measuring the cognitive aspect of SWB tend to include a compendium of measures—a single-item scale (represented by the single-item on OLS), a context-free scale such as the Students’ Life Satisfaction Scale (SLSS; Huebner, 1991); and domain-based scales such as the Brief Multidimensional Students’ Life Satisfaction Scale (BMSLSS; Seligson et al., 2003) and the Personal Well-Being Index-School Children (PWI-SC; Cummins and Lau, 2005). This strategy captures the breadth of the cognitive component of life satisfaction.

Given that PA and NA are key components of the SWB conceptual model, a common and often lamented practice (Busseri and Sadava, 2011) is researchers claiming to be investigating SWB, whilst only measuring life satisfaction. When affect is included, empirical initiatives have shown a preference for research on PA based on the assumption that it is more strongly associated with SWB (Casas and González-Carrasco, 2020), with fewer studies including measures of negative affect. PA pertains to the experience of positive emotions such as happiness, enthusiasm, and feeling energetic, and alert. NA refers to adverse states such as sadness, lethargy, boredom, and feeling stressed.

A major advancement in the literature on affect was made by Barrett and Russell (1998) (see also Russell, 2003) who introduced the concept of core affect—wherein affect is conceptualized on a circumflex, orthogonal two-dimensional model represented by pleasure-displeasure (pleasure or valence) and activation-deactivation (arousal or energy). Cummins (2014) harmonized the core affect components of happiness, contentment, and positive arousal into a single construct, which he refers to as Homeostatically Protected Mood (HPMood). There is a strong line of argument, backed by considerable evidence, which points to HPMood being the key component of SWB, which is also evident in adolescents (see Tomyn and Cummins, 2011; Cummins, 2014).

### The Current Study

Research into the structure of SWB has mostly included adult samples, with some exceptions (see e.g., Tomyn and Cummins, 2011). However, given the consistent finding from large-scale comparative studies that adults SWB and children’s SWB are independent of one another, children’s SWB should therefore be explored in its own right (see Rees, 2017; Rees et al., 2020). This represents the key rationale of the current study. We test the fit structure of a hierarchical structural (second-order) model of children’s SWB, which includes the context-free and domain-based cognitive components, and positive and negative affect. Research into the structure of SWB is primarily concerned with the structural relations between the various components. Given the multifaceted nature of the concept of SWB, we note Diener and Biswas-Diener’s (2000) contention that the simultaneous use of global (context-free) and specific (domain-based) measures strengthen our understanding of SWB, and more accurately captures the complexity of the concept. The primary goal of hierarchical second-order factor analysis is to provide a more parsimonious account of the correlations among lower-order factors, and is particularly useful for theory testing (Byrne, 2005; Brown, 2015). A hierarchical second-order model is premised on the notion that the first-order factors are highly correlated and that a single, second-order factor can account for the relations among the lower order factors. We premise our model on the theoretical proposition that a single higher-order factor of SWB manifests in four intercorrelated factors (cognitive context-free life satisfaction, cognitive domain-based life satisfaction, PA and NA). We hypothesize positive factor loadings between the second-order factor of SWB with the context-free and domain-based cognitive factors, and PA; and negative associations with NA. Given that the empirical feasibility of second-order analysis is based on the first-order factors being highly correlated, we first test a four-factor model of SWB to ascertain the strength of the intercorrelations. We hypothesize positive associations between the context-free, domain-based components, and PA; and negative correlations between the context-free, domain-based components, and NA. We also test the fit structure of the tripartite model of SWB, consisting of cognitive context-free life satisfaction, PA and NA. This allows for comparison with the four-factor model. Finally, given the differential experiences of well-being across age and gender, we also conduct measurement invariance testing to determine the comparability of the hierarchical model across these groups.

## Method

### Data Source

The study uses data from Wave 3 of the Children’s Worlds International Survey on Children’s Well-Being (see www.isciweb.org), which is the largest multinational study to assess children’s subjective perceptions and evaluations of their lives and well-being across different contexts and domains (Rees et al., 2020). Wave 3 of the survey was conducted in 35 countries from different regions and includes a school-based sample of 128,184 children in three age groups (8, 10, and 12-years-old). The analysis of the current study is limited to children in the 10- and 12-year-old age cohorts, yielding a total sample of *N* = 95,576. Given practical and resource constraints, the sampling frame for this wave of the survey was limited to mainstream schools. Each participating country was responsible for developing an individual country-specific sampling strategy, taking into consideration the characteristics of the school system and country-specific demographics. These strategies further aligned to prescribed criteria that included the specification of a probability sample of the target population of the defined geographical unit (some countries used country-wide samples, while others were limited to specific geographical regions), a minimum target sample size of 1,000 children in each age group, and a spread of schools to account for clustering. Ethics procedures were aligned to the country-specific requirements and each participating country obtained ethics clearance from Institutional Review Boards of the universities at which the researchers are based.

### Measures

How to measure children’s SWB is the one of the main issues identified in relation to research in the discipline. Traditionally, scales initially developed for use in adult samples were adapted for use with children. The Children’s Worlds Study is unique in that the measurement instruments used in the study reflect considerable engagement with children from various contexts in its development. Further to that, we were also able to incorporate advances made in previous psychometric studies that used data from previous waves of the survey (see Casas, 2017) to enhance the credibility of the measurement process, and to improve the cross-cultural comparability. Through these processes, we were able to construct specific measurement scales to assess the various components of children’s SWB.

Based on the above-mentioned advances, we present the Children’s Worlds Subjective Well-Being Scale (CW-SWBS), to measure context-free, cognitive life satisfaction. The scale consists of six items, three taken from the original SLSS and three new items proposed by children who participated in the qualitative research. The importance of including domain-based items in measuring SWB is well-established in the literature (Diener et al., 1999; Davern et al., 2007). Given the appropriateness of combining items of different levels of abstraction, we developed the Children’s Worlds: Domain-Based Subjective Well-Being Scale (CW-DBSWBS). The scale comprises 11-items that assess children’s domain-based cognitive life satisfaction, with each item representing a particular life domain. The scale was developed using the five concretely worded items from the BMSLSS (Seligson et al., 2003), namely: family (people children live with), friends, school (life as a student), area/environment (the place where children live), and self (the way you look); and four items from the abstractly worded PWI-SC (Cummins and Lau, 2005), namely: standard of living (things you have), personal health, personal safety, and future security. We included two extra items found to make an important contribution to children’s lives; one on satisfaction with time-use (Casas et al., 2013) and another on satisfaction with freedom (Rees, 2017). Participants were asked to rate their satisfaction on an 11-point unipolar satisfaction scale ranging from “Not at all” (0) to “Totally/Completely satisfied” (10).

Six items drawn from Russel’s Model of Core Affect were included in the survey. These items specify a concrete time-period (2 weeks) wherein participants are asked to endorse the extent to which they experienced three positive (happy, calm, and full of energy) and three negative (sad, stressed, and bored) affective states. For both PA and NA, these items reflect a pleasant-unpleasant, activated-deactivated and neutral affect (see Rees, 2019). Given the specified time-period, the scale is purported to measure state level affective well-being. While Feist et al. (1995) notes that SWB can be conceptualized as a trait and state variable, there is a broad debate in relation to whether affective well-being should be considered more as state or trait variable (see Schimmack, 2007). Research conducted by Jovanovich (2015) on the structure of SWB using the PANAS found that measuring affective SWB at the trait or state level did not impact on the structure of SWB, which included trait level measurement of cognitive well-being.

Finally, to test convergent validity, we included the single-item on OLS (Cummins and Lau, 2005) using the following wording: How satisfied are you with your life as a whole? Campbell et al. (1976) and Cummins and Lau (2005), affirm the importance of including a single-item OLS as a means to test the convergent validity of SWB scales. Casas and Rees (2015), and Casas (2017) similarly note its applicability for ascertaining convergent validity of children’s SWB scales.

### Data Analytic Strategy

The final Children’s Worlds database consisted of 95,576 participants. The cleaning and depuration of the dataset followed a set of procedures that included conducting a missing data analysis, assessing the presence of systematic response sets, and attending to clustering as an outcome of survey design effects. In the current study, we found missing items to be “missing completely at random” (MCAR) (Little’s MCAR: *X^2^* = 319.453, *df* = 282, *p* = 0.062). We adopted a broad strategy for attending to missing data, ensuring that for each relevant scale only cases with less than 50% missing were subject to missing data estimation, and excluding those with more than 50% missing. For the estimation of the missing data, we used full information maximum likelihood (FIML), a modern approach to estimation, which has shown to produce unbiased parameter estimates and standard errors when data are MCAR (Enders, 2010). After data cleaning and depuration of the dataset, the overall sample of the Children’s Worlds Study was reduced by 2,794 cases to yield a final sample for the current study of 92,782 participants (girls = 49.7%; *M*_age_ = 11.03; *SD* = 1.27). Our data analysis strategy followed a three-step process as recommended by Brown (2015). First, we tested the conceptual and empirical viability of a four-factor model. Second, we examined the strength and pattern of correlations among factors in the first-order model. Third, we presented and tested the fit structure of the second-order factor model—justified on theoretical and empirical grounds.

### Data Analysis

Data were analyzed using confirmatory factor analysis in AMOS 26. Following the recommendations by Jackson et al. (2009), we used the Comparative Fit Index (CFI), the Standardized Root Mean Residual (SRMR), and the Root Mean Squared Error of Approximation (RMSEA) as fit indices to assess model fit. For these fit indices, we followed the cut-off thresholds recommended by Casas (2017). For the CFI we accepted scores higher than 0.950, and for the SRMR and the RMSEA we accepted scores lower than 0.05 as indicators of a good fit.

We conducted measurement invariance testing, using multi-group structural equation modeling (MGSEM), to test the comparability of the conceptual model across gender and age. The tenability of measurement invariance allows for meaningful and unambiguous group comparisons (Meredith, 1993). We achieved this by following a three-step sequential process comprising the application of incrementally restrictive constraints wherein configural, metric, and scalar invariance were tested. Each subsequent constrained model was regarded as tenable if the model fit did not worsen by more than 0.01 on the CFI (Cheung and Rensvold, 2002) and by 0.015 on the RMSEA and SRMR (Chen, 2007). The tenability of scalar measurement invariance indicates that groups (gender and age) are comparable by correlations, regression coefficients, and mean scores.

## Results

Skewness and kurtosis of the items on all the scales fell beyond acceptable thresholds (Finney and DiStefano, 2006; see Table 1). We used the bootstrap method (500 samples) in AMOS 26 to attend to the deviation (Blunch, 2008). The bootstrap procedure allows for more accurate parameter estimates and the efficient handling of standard errors in the context of non-normal data (Blunch, 2008; Enders, 2010; Brown, 2015; Casas and González, 2017). A reliability analysis demonstrated acceptable reliability coefficients of 0.93 for the CW-SWBS, 0.87 for the CW-DBSWBS, 0.60 for PA scale, and 0.68 for the NA scale. We present the descriptive statistics for the items presented in Table 1 and the item correlations in Table 2.

**TABLE 1:** Item means and standard deviations.

Item	Mean	SD	Skewness	Kurtosis
**CW-SWBS**				
I enjoy life	8.757	2.050	–2.096	7.424
My life is going well	8.629	2.150	–1.941	6.655
I have good life	8.798	2.044	–2.180	7.749
Things in my life are excellent	8.159	2.404	–1.152	18.273
I like my life	8.819	2.100	–2.248	7.946
I am happy with my life	8.837	2.094	–2.290	8.150
**CW-DBSWBS**				
Satisfied with the people you live with	8.941	1.889	–2.289	8.466
Satisfied with your life as student	8.440	2.131	–1.755	6.107
Satisfied with your friends	8.563	2.066	–1.865	6.482
Satisfied with your local area	8.416	2.264	–1.835	6.167
Satisfied with the things have	8.906	1.877	–2.329	8.934
Satisfied with how you use your time	8.426	2.085	–1.712	6.020
Satisfied with your safety	8.816	1.912	–2.157	8.067
Satisfied with your freedom	8.496	2.198	–1.857	6.293
Satisfied with how you look (appearance)	8.299	2.414	–1.689	5.379
Satisfied with what may happen to you later in life	8.390	2.272	–1.833	6.203
Satisfied with your health	8.928	1.904	–2.354	8.888
**CW-PA**				
Feeling happy	8.628	2.078	–1.963	6.887
Feeling calm	7.362	2.859	–1.035	3.190
Feeling full of energy	8.145	2.684	–1.605	4.765
**CW-NA**				
Feeling sad	3.620	3.280	0.566	2.072
Feeling stressed	4.139	3.603	0.292	1.650
Feeling bored	4.341	3.534	0.255	1.701

**TABLE 2:** Correlation matrix for items on the scales.

Item	Enjoy life	Life going well	Have good life	Things life excellent	Like my life	Happy with my life	Satisfied life as student	Satisfied friends	Satisfied local area	Satisfied things have	Satisfied time use	Satisfied safety	Satisfied freedom	Satisfied appearance	Satisfied later in life	Satisfied health	Satisfied people live with	Feeling happy	Feeling sad	Feeling calm	Feeling stressed	Feeling full of energy	Feeling bored
Enjoy life	1.00																						
Life going well	0.70	1.00																					
Have good life	0.70	0.76	1.00																				
Things life excellent	0.60	0.65	0.65	1.00																			
Like my life	0.70	0.71	0.73	0.64	1.00																		
Happy with my life	0.69	0.71	0.72	0.64	0.80	1.00																	
Satisfied life as student	0.44	0.46	0.43	0.41	0.44	0.44	1.00																
Satisfied friends	0.40	0.35	0.34	0.33	0.33	0.33	0.33	1.00															
Satisfied local area	0.37	0.38	0.37	0.35	0.37	0.38	0.31	0.31	1.00														
Satisfied things have	0.43	0.43	0.44	0.39	0.43	0.43	0.31	0.32	0.39	1.00													
Satisfied time use	0.45	0.46	0.45	0.42	0.44	0.45	0.36	0.33	0.37	0.48	1.00												
Satisfied safety	0.49	0.49	0.50	0.45	0.48	0.48	0.37	0.34	0.40	0.46	0.46	1.00											
Satisfied freedom	0.46	0.47	0.47	0.44	0.46	0.46	0.33	0.30	0.35	0.44	0.46	0.51	1.00										
Satisfied appearance	0.44	0.45	0.44	0.42	0.45	0.45	0.35	0.28	0.30	0.37	0.41	0.45	0.44	1.00									
Satisfied later in life	0.42	0.44	0.44	0.43	0.43	0.43	0.34	0.28	0.31	0.37	0.39	0.45	0.44	0.46	1.00								
Satisfied health	0.41	0.43	0.43	0.38	0.42	0.42	0.32	0.28	0.31	0.39	0.39	0.46	0.44	0.46	0.42	1.00							
Satisfied people live with	0.43	0.44	0.44	0.39	0.43	0.43	0.36	0.32	0.32	0.40	0.38	0.41	0.38	0.32	0.32	0.34	1.00						
Feeling happy	0.51	0.52	0.51	0.47	0.52	0.54	0.36	0.31	0.32	0.39	0.42	0.45	0.43	0.42	0.39	0.40	0.36	1.00					
Feeling sad	-0.20	-0.22	-0.21	-0.19	-0.22	-0.22	-0.11	-0.13	-0.10	-0.14	-0.15	-0.17	-0.16	-0.16	-0.13	-0.15	-0.14	-0.22	1.00				
Feeling calm	0.27	0.30	0.28	0.27	0.28	0.29	0.24	0.17	0.21	0.22	0.26	0.26	0.25	0.27	0.25	0.24	0.19	0.35	-0.02	1.00			
Feeling stressed	-0.17	-0.19	-0.17	-0.17	-0.17	-0.18	-0.14	-0.10	-0.10	-0.10	-0.13	-0.13	-0.14	-0.15	-0.13	-0.13	-0.11	-0.16	0.43	-0.08	1.00		
Feeling full of energy	0.30	0.31	0.30	0.29	0.30	0.32	0.25	0.20	0.22	0.23	0.26	0.27	0.24	0.29	0.27	0.28	0.21	0.40	-0.07	0.27	-0.08	1.00	
Feeling bored	-0.15	-0.16	-0.16	-0.15	-0.16	-0.16	-0.12	-0.10	-0.10	-0.10	-0.13	-0.12	-0.13	-0.13	-0.11	-0.11	-0.10	-0.14	0.42	-0.03	0.39	-0.06	1.00

*All correlation coefficients significant at p < 0.001. The correlation matrix was generated with Stata 14.2 using (pwcorr) with Bonferroni correction to mitigate Type I error.*

### Confirmatory Factor Analysis

**FIGURE 1:**
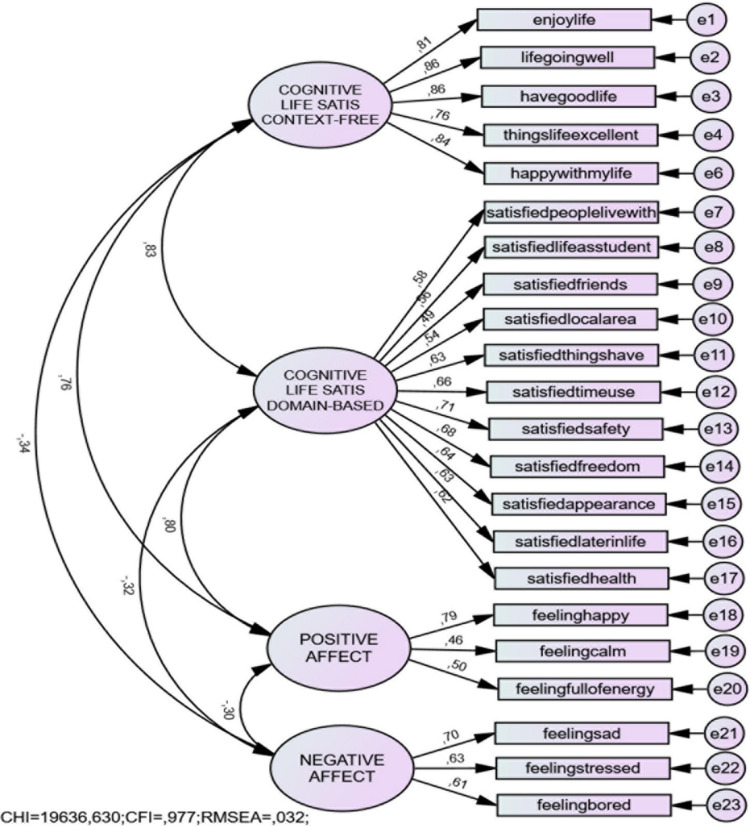
Four-factor confirmatory factor model.

We commenced the CFA by testing the fit structure of the individual scales, all of which presented with an appropriate fit (see Models 1–3 in Table 3). Thereafter, as per the aforementioned data analytic strategy, we specified a multiple factor model, in line with the traditional conceptualization of SWB. However, for the cognitive component, we specified two distinct factors, one representing context-free life satisfaction, and another representing domain-based life satisfaction (see Model 4 in Table 3). The fit statistics of this model met the thresholds for a good fit, with predicted positive correlations between the life satisfaction constructs and PA, and negative correlations between NA and life satisfaction constructs and PA (see Figure 1). We noted shared variance between the two items “I like my life” and “I am happy with my life” in the context-free cognitive life satisfaction latent variable. While the inclusion of an error covariance between these two items improved the model fit (see Model 5 in Table 3), we found a high correlation between the errors (0.32). This finding suggests the presence of method bias, which is likely due to similarity in the wording of the items. The participants may be understanding the content of the items in the same way. Excluding the item “I like my life” improved the fit of the initial model and provided the best statistical and theoretical fit to the data (Model 6 in Table 3). All standardized regression weights between the latent constructs and the indicator variables were significant (see Table 3), suggesting that all the items were reasonable indicators of their respective factors. Furthermore, the strength of the correlations provided the empirical justification for pursuing the testing of a second-order factor.

**TABLE 3:** Fit indices for the confirmatory factor and structural equation models.

Model Bootstrap, ML, 95% confidence intervals, resamples = 500	Chi-square	*df*	Sig	CFI	RMSEA	SRMR
1. Model 1: CW-SWBS	7571.125	9	0.000	0.982	0.052 (0.049–0.055)	0.017
2. Model 2: CW-DBSWBS	9619.482	44	0.000	0.969	0.049 (0.048–0.049)	0.027
3. Model 3: PA/NA	2080.564	8	0.000	0.974	0.034 (0.051–0.055)	0.028
4. Model 4: CFA 4-factor	27154.902	224	0.000	0.972	0.036 (0.036–0.036	0.022
5. Model 5: CFA 4-factor model with 1 error covariance	20244.453	223	0.000	0.979	0.031 (0.031–0.032)	0.021
6. Model 6: CFA 4-factor model with “I like my life” excluded.	19636.630	203	0.000	0.977	0.032 (0.032–0.033)	0.021
7. Model 7: hierarchical second-order model with 1 ECV	20414.258	225	0.000	0.979	0.031 (0.031–0.032)	0.021
8. Model 8: hierarchical second-order model (final model with “I like my life excluded”)	20036.426	205	0.000	0.977	0.032 (0.032–0.33)	0.021
9. Model 9: hierarchical second-order model: configural invariance (age)	21753.486	410	0.00	0.975	0.024 (0.023–0.24)	0.021
10. Model 10: hierarchical second-order model: metric invariance (age)	22172.685	428	0.00	0.974	0.023 (0.023–0.024)	0.022
11. Model 11: hierarchical second-order model: scalar invariance (age)	23366.463	446	0.00	0.973	0.023 (0.023–0.024	0.022
12. Model 12: hierarchical second-order model: configural invariance (gender)	20229.059	410	0.00	0.976	0.023 (0.023–0.023)	0.022
13. Model 13: hierarchical second-order model: metric invariance (gender)	20455.244	428	0.00	0.976	0.023 (0.022–0.023)	0.022
14. Model 14: hierarchical second-order model: scalar invariance (gender)	21879.180	446	0.00	0.974	0.023 (0.023–0.023)	0.022
15. Model 15: hierarchical second-order tripartite model	3822.530	41	0.00	0.992	0.031 (0.030–0.032)	0.018

### Second-Order Hierarchical Structural Equation Modeling

**FIGURE 2:**
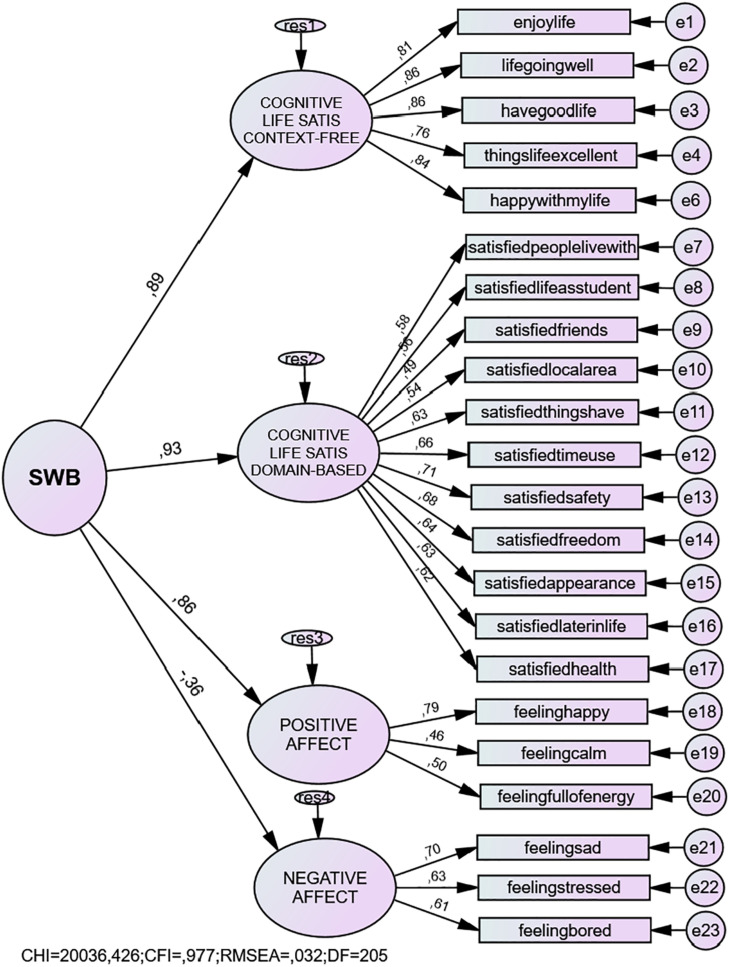
Hierarchical structural model of children’s subjective well-being.

Given the nature of the intercorrelations between the four latent factors, we proceeded to test second-order hierarchical models. For this analysis we tested two models; one which included an error covariance between the items “I like my life” and “I am happy with my life” (see Model 7 in Table 3), and another with “I like my life” excluded (see Model 8 in Table 3; Figure 2). While we observed stronger fit statistics for the former, the factor loadings of the second-order latent constructs on the higher-order construct were identical. We found strong loadings of the second-order latent construct (SWB) on the components in the anticipated direction, indicating that the latent SWB factor explains a substantial amount of variance in each of the specified components (see Table 4; Figure 2). We found substantial positive loadings for both life satisfaction components and for PA—the highest loading was for the domain-based cognitive life satisfaction component (0.93); while the lowest was reflected in a moderate negative loading with NA (−0.36). We also tested the viability of a tripartite hierarchical model with three first-order factors (context-free cognitive life satisfaction, positive and negative affect) (see Model 15 in Table 3). Here we found that this model presented with a better overall fit than the hierarchical model with four first-order factors. Finally, we also tested convergent validity by regressing the model onto the observed variable OLS. We found a standardized regression weight of 0.77, which indicates acceptable convergent validity.

**TABLE 4:** Standardized regression weights: second-order hierarchical model.

Parameter Bootstrap, ML, 95% confidence intervals, resamples = 500			Estimate	Lower	Upper
CWSWBS	←	SWB	0.893	0.888	0.898
Positive_Affect	←	SWB	0.859	0.852	0.866
Negative_Affect	←	SWB	–0.358	–0.366	–0.350
DOMAINSWB	←	SWB	0.929	0.924	0.935
enjoylife	←	CWSWBS	0.814	0.810	0.820
lifegoingwell	←	CWSWBS	0.861	0.857	0.865
havegoodlife	←	CWSWBS	0.862	0.858	0.866
thingslifeexcellent	←	CWSWBS	0.759	0.748	0.770
happywithmylife	←	CWSWBS	0.840	0.835	0.844
satisfiedpeoplelivewith	←	DOMAINSWB	0.577	0.570	0.585
satisfiedlifestudent	←	DOMAINSWB	0.558	0.550	0.566
satisfiedfriends	←	DOMAINSWB	0.488	0.481	0.496
satisfiedlocalarea	←	DOMAINSWB	0.538	0.531	0.545
satisfiedthingshave	←	DOMAINSWB	0.632	0.625	0.639
satisfiedtimeuse	←	DOMAINSWB	0.660	0.654	0.667
satisfiedsafety	←	DOMAINSWB	0.712	0.706	0.718
satisfiedfreedom	←	DOMAINSWB	0.677	0.671	0.683
satisfiedappearance	←	DOMAINSWB	0.638	0.632	0.645
satisfiedlaterinlife	←	DOMAINSWB	0.626	0.619	0.633
satisfiedhealth	←	DOMAINSWB	0.622	0.615	0.628
feelinghappy	←	Positive Affect	0.794	0.787	0.801
feelingcalm	←	Positive Affect	0.456	0.448	0.464
feelingfullofenergy	←	Positive Affect	0.501	0.493	0.510
feelingsad	←	Negative Affect	0.697	0.690	0.704
feelingstressed	←	Negative Affect	0.630	0.623	0.638
feelingbored	←	Negative Affect	0.608	0.601	0.615

*All items significant at p < 0.01. Confidence intervals generated in Stata 14.2.*

### Multi-Group Analysis

To determine the comparability of the model across age and gender, we conducted measurement invariance testing using MGSEM. The process entailed testing configural, metric and scalar invariance through the application of increasingly restrictive constraints. As depicted in Models 9 to 14, the reduction in fit fell within the acceptable criteria of not worsening by more than 0.01 on the CFI, nor by 0.015 on the RMSEA and SRMR (Cheung and Rensvold, 2002; Chen, 2007). Configural, metric and scalar measurement invariance were thus attained—this suggests that the model is comparable across age and gender by correlation and regression coefficients and mean scores. The standardized regression weights of the configural, metric, and scalar measurement invariance models are presented in Tables 5–7. Across age, the loadings for the 12-year-olds were slightly higher than the 10-year-olds, while across gender we found higher loadings for girls than boys. Across all the subgroups, the second-order construct of SWB loaded more strongly onto the domain-based latent construct.

**TABLE 5:** Standardized regression weights: configural model with unconstrained loadings (age and gender).

			10-year-olds			12-year-olds			Boys			Girls		
Parameter Bootstrap, ML, 95% Confidence Intervals, Resamples = 500			Estimate	Lower	Upper	Estimate	Lower	Upper	Estimate	Lower	Upper	Estimate	Lower	Upper
CWSWBS	←	SWB	0.878	0.870	0.885	0.904	0.897	0.910	0.880	0.873	0.888	0.900	0.893	0.905
Positive Affect	←	SWB	0.852	0.841	0.863	0.862	0.852	0.871	0.855	0.843	0.866	0.865	0.856	0.874
Negative_Affect	←	SWB	–0.301	–0.314	–0.289	–0.413	–0.425	–0.402	–0.319	–0.333	–0.307	–0.388	–0.400	–0.378
DOMAINSWB	←	SWB	0.920	0.910	0.930	0.938	0.930	0.944	0.924	0.917	0.934	0.938	0.931	0.946
enjoylife	←	CWSWBS	0.783	0.775	0.792	0.840	0.834	0.847	0.797	0.789	0.806	0.828	0.820	0.834
lifegoingwell	←	CWSWBS	0.838	0.830	0.844	0.880	0.875	0.886	0.846	0.839	0.851	0.872	0.866	0.877
havegoodlife	←	CWSWBS	0.841	0.834	0.848	0.881	0.875	0.886	0.848	0.841	0.855	0.874	0.868	0.879
thingslifeexcellent	←	CWSWBS	0.735	0.726	0.743	0.780	0.753	0.794	0.736	0.705	0.750	0.777	0.770	0.784
happywithmylife	←	CWSWBS	0.812	0.804	0.818	0.862	0.857	0.866	0.823	0.816	0.829	0.852	0.846	0.858
satisfiedpeoplelivewith	←	DOMAINSWB	0.559	0.547	0.569	0.597	0.586	0.606	0.563	0.553	0.575	0.594	0.582	0.605
satisfiedlifeasstudent	←	DOMAINSWB	0.544	0.534	0.557	0.567	0.556	0.577	0.532	0.521	0.542	0.590	0.580	0.601
satisfiedfriends	←	DOMAINSWB	0.498	0.488	0.510	0.476	0.465	0.486	0.499	0.488	0.511	0.482	0.472	0.493
satisfiedlocalarea	←	DOMAINSWB	0.527	0.516	0.537	0.540	0.531	0.550	0.529	0.518	0.539	0.553	0.543	0.562
satisfiedthingshave	←	DOMAINSWB	0.628	0.616	0.638	0.630	0.620	0.640	0.636	0.624	0.645	0.631	0.619	0.640
satisfiedtimeuse	←	DOMAINSWB	0.647	0.636	0.656	0.666	0.657	0.674	0.654	0.644	0.663	0.664	0.655	0.673
satisfiedsafety	←	DOMAINSWB	0.706	0.697	0.716	0.716	0.707	0.724	0.718	0.710	0.727	0.717	0.707	0.725
satisfiedfreedom	←	DOMAINSWB	0.667	0.659	0.677	0.686	0.677	0.695	0.673	0.663	0.682	0.680	0.671	0.688
satisfiedappearance	←	DOMAINSWB	0.621	0.611	0.630	0.649	0.641	0.658	0.635	0.626	0.644	0.640	0.632	0.650
satisfiedlaterinlife	←	DOMAINSWB	0.592	0.583	0.604	0.659	0.650	0.669	0.619	0.609	0.629	0.631	0.622	0.641
satisfiedhealth	←	DOMAINSWB	0.609	0.598	0.620	0.631	0.621	0.641	0.627	0.615	0.637	0.617	0.607	0.625
feelinghappy	←	Positive_Affect	0.770	0.760	0.781	0.813	0.804	0.821	0.784	0.774	0.793	0.796	0.787	0.804
feelingcalm	←	Positive_Affect	0.446	0.437	0.457	0.464	0.455	0.475	0.438	0.427	0.449	0.469	0.458	0.479
feelingfullofenergy	←	Positive_Affect	0.468	0.456	0.480	0.521	0.510	0.532	0.504	0.492	0.515	0.536	0.525	0.547
feelingsad	←	Negative_Affect	0.692	0.683	0.703	0.701	0.691	0.711	0.686	0.675	0.697	0.704	0.694	0.715
feelingstressed	←	Negative_Affect	0.640	0.631	0.649	0.621	0.611	0.631	0.634	0.624	0.645	0.626	0.616	0.635
feelingbored	←	Negative_Affect	0.628	0.619	0.638	0.581	0.572	0.592	0.620	0.609	0.631	0.604	0.596	0.614

*All items significant at p < 0.01; confidence intervals generated in Stata 14.2.*

**TABLE 6:** Standardized regression weights: metric model with constrained loadings (age and gender).

			10-year-olds			12-year-olds			Boys			Girls		
Parameter Bootstrap, ML, 95% confidence intervals, resamples = 500			Estimate	Lower	Upper	Estimate	Lower	Upper	Estimate	Lower	Upper	Estimate	Lower	Upper
CWSWBS	←	SWB	0.878	0.870	0.886	0.904	0.897	0.909	0.880	0.873	0.888	0.900	0.893	0.906
Positive Affect	←	SWB	0.851	0.840	0.862	0.862	0.853	0.872	0.857	0.845	0.868	0.863	0.855	0.873
Negative_Affect	←	SWB	–0.301	–0.314	–0.289	–0.413	–0.424	–0.401	–0.319	–0.333	–0.307	–0.388	–0.400	–0.378
DOMAINSWB	←	SWB	0.920	0.910	0.930	0.938	0.930	0.945	0.925	0.917	0.934	0.937	0.930	0.945
enjoylife	←	CWSWBS	0.786	0.777	0.793	0.839	0.833	0.845	0.798	0.791	0.807	0.827	0.820	0.833
lifegoingwell	←	CWSWBS	0.836	0.829	0.842	0.881	0.876	0.887	0.844	0.838	0.850	0.873	0.867	0.878
havegoodlife	←	CWSWBS	0.836	0.830	0.843	0.884	0.878	0.888	0.846	0.839	0.852	0.876	0.870	0.880
thingslifeexcellent	←	CWSWBS	0.730	0.722	0.737	0.782	0.757	0.797	0.736	0.706	0.750	0.776	0.770	0.784
happywithmylife	←	CWSWBS	0.819	0.814	0.825	0.857	0.851	0.861	0.826	0.820	0.832	0.850	0.844	0.856
satisfiedpeoplelivewith	←	DOMAINSWB	0.557	0.547	0.567	0.599	0.590	0.607	0.561	0.551	0.569	0.596	0.586	0.605
satisfiedlifeasstudent	←	DOMAINSWB	0.546	0.537	0.556	0.565	0.556	0.573	0.532	0.524	0.540	0.532	0.524	0.540
satisfiedfriends	←	DOMAINSWB	0.477	0.469	0.486	0.497	0.489	0.506	0.491	0.483	0.500	0.491	0.483	0.500
satisfiedlocalarea	←	DOMAINSWB	0.528	0.519	0.537	0.539	0.531	0.548	0.532	0.523	0.540	0.532	0.523	0.540
satisfiedthingshave	←	DOMAINSWB	0.627	0.619	0.637	0.631	0.623	0.640	0.624	0.615	0.633	0.624	0.615	0.633
satisfiedtimeuse	←	DOMAINSWB	0.652	0.643	0.660	0.661	0.653	0.668	0.654	0.645	0.661	0.654	0.645	0.661
satisfiedsafety	←	DOMAINSWB	0.700	0.692	0.709	0.721	0.714	0.729	0.718	0.710	0.725	0.718	0.710	0.725
satisfiedfreedom	←	DOMAINSWB	0.660	0.653	0.669	0.692	0.685	0.700	0.675	0.667	0.683	0.675	0.667	0.683
satisfiedappearance	←	DOMAINSWB	0.632	0.625	0.640	0.637	0.629	0.646	0.650	0.643	0.658	0.650	0.643	0.658
satisfiedlaterinlife	←	DOMAINSWB	0.599	0.591	0.609	0.653	0.646	0.662	0.624	0.614	0.632	0.624	0.614	0.632
satisfiedhealth	←	DOMAINSWB	0.613	0.606	0.622	0.626	0.618	0.634	0.619	0.610	0.629	0.624	0.616	0.631
feelinghappy	←	Positive_Affect	0.772	0.763	0.781	0.811	0.803	0.819	0.777	0.768	0.786	0.777	0.768	0.786
feelingcalm	←	Positive_Affect	0.424	0.415	0.434	0.481	0.473	0.490	0.440	0.431	0.449	0.440	0.431	0.449
feelingfullofenergy	←	Positive_Affect	0.482	0.473	0.491	0.509	0.500	0.518	0.517	0.508	0.526	0.517	0.508	0.526
feelingsad	←	Negative_Affect	0.701	0.694	0.710	0.691	0.683	0.699	0.695	0.687	0.703	0.695	0.687	0.703
feelingstressed	←	Negative_Affect	0.639	0.632	0.647	0.622	0.614	0.629	0.631	0.623	0.639	0.631	0.623	0.639
feelingbored	←	Negative_Affect	0.617	0.609	0.624	0.594	0.587	0.603	0.612	0.603	0.619	0.612	0.603	0.619

*All items significant at p < 0.01; confidence intervals generated in Stata 14.2.*

**TABLE 7:** Standardized regression weights: scalar model with constrained loadings and intercepts (age and gender).

			10-year-olds			12-year-olds			Boys			Girls		
Parameter Bootstrap, ML, 95% confidence intervals, resamples = 500			Estimate	Lower	Upper	Estimate	Lower	Upper	Estimate	Lower	Upper	Estimate	Lower	Upper
CWSWBS	←	SWB	0.878	0.871	0.887	0.903	0.897	0.910	0.879	0.873	0.888	0.900	0.893	0.906
Positive Affect	←	SWB	0.856	0.842	0.864	0.865	0.855	0.874	0.861	0.845	0.868	0.866	0.855	0.873
Negative_Affect	←	SWB	–0.303	–0.317	–0.292	–0.410	–0.428	–0.405	–0.320	–0.333	–0.308	–0.387	–0.400	–0.379
DOMAINSWB	←	SWB	0.921	0.911	0.931	0.939	0.931	0.945	0.927	0.917	0.934	0.938	0.930	0.945
enjoylife	←	CWSWBS	0.786	0.778	0.794	0.839	0.835	0.847	0.799	0.791	0.807	0.826	0.820	0.833
lifegoingwell	←	CWSWBS	0.837	0.830	0.843	0.882	0.877	0.888	0.846	0.838	0.850	0.873	0.867	0.878
havegoodlife	←	CWSWBS	0.837	0.830	0.843	0.884	0.879	0.889	0.848	0.839	0.852	0.876	0.870	0.880
thingslifeexcellent	←	CWSWBS	0.732	0.724	0.739	0.783	0.760	0.799	0.738	0.706	0.750	0.776	0.771	0.784
happywithmylife	←	CWSWBS	0.820	0.815	0.826	0.858	0.853	0.863	0.827	0.820	0.832	0.850	0.844	0.856
satisfiedpeoplelivewith	←	DOMAINSWB	0.555	0.547	0.566	0.596	0.590	0.607	0.561	0.551	0.569	0.595	0.586	0.604
satisfiedlifeasstudent	←	DOMAINSWB	0.546	0.540	0.558	0.566	0.560	0.576	0.528	0.521	0.538	0.588	0.580	0.596
satisfiedfriends	←	DOMAINSWB	0.476	0.470	0.487	0.496	0.490	0.507	0.492	0.483	0.500	0.491	0.482	0.499
satisfiedlocalarea	←	DOMAINSWB	0.530	0.522	0.541	0.541	0.536	0.552	0.532	0.522	0.539	0.550	0.542	0.559
satisfiedthingshave	←	DOMAINSWB	0.630	0.621	0.639	0.632	0.626	0.643	0.626	0.615	0.633	0.641	0.631	0.648
satisfiedtimeuse	←	DOMAINSWB	0.657	0.647	0.664	0.663	0.657	0.672	0.656	0.645	0.661	0.664	0.655	0.672
satisfiedsafety	←	DOMAINSWB	0.699	0.692	0.709	0.720	0.715	0.730	0.718	0.710	0.725	0.718	0.710	0.725
satisfiedfreedom	←	DOMAINSWB	0.659	0.653	0.669	0.691	0.686	0.701	0.677	0.667	0.683	0.678	0.671	0.686
satisfiedappearance	←	DOMAINSWB	0.634	0.628	0.643	0.639	0.633	0.650	0.650	0.643	0.658	0.623	0.616	0.632
satisfiedlaterinlife	←	DOMAINSWB	0.598	0.592	0.609	0.653	0.647	0.663	0.624	0.614	0.632	0.625	0.619	0.636
satisfiedhealth	←	DOMAINSWB	0.613	0.607	0.623	0.626	0.620	0.636	0.619	0.610	0.629	0.624	0.616	0.631
feelinghappy	←	Positive_Affect	0.771	0.765	0.783	0.810	0.805	0.821	0.778	0.768	0.786	0.800	0.794	0.809
feelingcalm	←	Positive_Affect	0.426	0.417	0.436	0.481	0.476	0.492	0.442	0.431	0.449	0.468	0.458	0.476
feelingfullofenergy	←	Positive_Affect	0.487	0.477	0.496	0.512	0.506	0.524	0.519	0.508	0.526	0.524	0.514	0.535
feelingsad	←	Negative_Affect	0.703	0.694	0.711	0.692	0.684	0.700	0.699	0.688	0.704	0.698	0.686	0.703
feelingstressed	←	Negative_Affect	0.636	0.631	0.646	0.620	0.613	0.628	0.628	0.623	0.640	0.627	0.621	0.637
feelingbored	←	Negative_Affect	0.618	0.610	0.626	0.596	0.588	0.605	0.611	0.603	0.619	0.611	0.605	0.620

*All items significant at p < 0.01; Confidence intervals generated in Stata 14.2.*

### Latent Variable Means Analysis

Given the tenability of scalar invariance, we proceeded to conduct a means analysis of the latent variables within the SEM framework, using the structured means analysis approach (see Supplementary Tables 1–4). Across age, using the 10-year-olds as the reference group, we found that the 12-year-olds scored significantly lower on the cognitive context-free latent variable (*M*_*diff*_ = −0.308, *p* < 0.001, Cohens *d* = 0.181, 95% CI [0.168, 0.194]), the cognitive domain-based life-satisfaction latent variable (*M*_*diff*_ = −0.253, *p* < 0.001, Cohens *d* = 0.199, 95% CI [0.186, 0.212]), and PA (*M*_*diff*_ = −0.398, *p* < 0.001, Cohens *d* = 0.203, 95% CI [0.190, 0.216]). On the NA latent variable, the 12-year-olds scored significantly higher (*M*_*diff*_ = 0.246, *p* < 0.001, Cohens *d* = −0.090, 95% CI [−0.103, −0.078]).

Across gender, using boys as the reference point, girls scored significantly lower on the cognitive context-free latent variable (*M*_*diff*_ = −0.056, *p* < 0.001, Cohens *d* = 0.034, 95% CI [0.021, 0.046]) and the PA latent variable (*M*_*diff*_ = −0.090, *p* < 0.001, Cohens *d* = 0.058, 95% CI [0.044, 0.071]). Girls scored significantly higher on the NA latent variable (*M*_*diff*_ = −0.258, *p* < 0.001, Cohens *d* = −0.085, 95% CI [−0.098, −0.072]). We found no significant difference on the cognitive domain-based life satisfaction variable.

## Discussion and Conclusion

The study aimed to test a model of children’s SWB, which includes the context-free and domain-based cognitive components of life satisfaction, PA, and NA. The study further aimed to ascertain the fit structure of a hierarchical structural (second-order) model of children’s SWB.

We found a good fit for the four-factor confirmatory factor model of children’s SWB. Correlations between the various latent factors were as anticipated—with positive correlations between the life satisfaction components and PA, and negative correlations with NA. We further found a good fit for the hierarchical structural model of children’s SWB (with both three and four first-order factors), which aligns to predictions made by Casas (2017). A considerable amount of variance in each indicator variable was accounted for by the second-order latent construct of SWB. This finding represents the key contribution of the study, and confirms the viability of a hierarchical structural conceptualization of children’s SWB. Ultimately, it lends support to the aforementioned theoretical assertion of Busseri (2018), and provides empirical evidence that extends the generalizability of the hierarchical conceptual model to child samples. Our theoretical proposition that a single higher-order factor of SWB manifests in four intercorrelated lower-order factors (cognitive context-free life satisfaction, cognitive domain-based life satisfaction, PA, and NA) is thus supported. Further to that, the viability of the model creates opportunities to explore correlates and predictors of children’s SWB using the full conceptual model. Importantly, we note that the inclusion of the domain-based latent construct contributes to the overall fit structure of the model, and propound the tenability of a quadripartite hierarchical conceptual model of children’s SWB. This finding aligns to the findings of previous studies on adult SWB conducted by Galinha and Pais-Ribeiro (2009) who found appropriate fit structure for models that included both global and specific measures of SWB. The key advantage of using multiple measures (global and specific) of SWB is that it gives a higher level of certainty to the results and increases our understanding of the underlying factors impacting on SWB (Diener and Biswas-Diener, 2000). In the current study, we found that children’s SWB manifested more strongly in the domain-based component (latent construct) than any of the other components. Taking the above into consideration, we therefore recommend the theoretical supposition of the quadripartite model (including the domain-based component) even though the tripartite model presented with stronger fit indices.

We note the shared variance between the items “I like my life” and “I am happy with my life” on the CW-SWBS. This overlap is likely due to method variance or in the similarity of the wording of the items. We considered the inclusion of an error covariance, and the deletion of the item “I like my life”—with both options resulting in the improvement of model fit. In the context of the current study, we nominate the latter as the final model (with the item “I like my life” excluded). Our motivation is that this model presents as more parsimonious and maximizes its potential for developing more complex structural equation models. We recommend that future research take cognizance of this finding when developing models that are more complex or when exploring the relation between SWB and other variables. We used MGSEM to test the comparability of the hierarchical model across age and gender. Our results confirm the tenability of scalar invariance across age and gender. This finding indicates that the items of the model are understood in the same way across these groups, and meaningful comparison across correlations, regressions, and means are viable. Across age, we found significant differences for the four latent constructs; and across gender we found significant differences for all the latent constructs, except the cognitive domain-based latent construct. However, we note small effect sizes. Given that the focus of the current study is on the structural relations between the various components, our interest was comparing the manifestation (loadings) of the second-order construct of SWB onto the cognitive and affective latent constructs. Across age, we found slightly stronger loadings of SWB onto the four latent constructs for the 12-years-olds than for the 10-year-olds; across gender, we found slightly stronger loadings for girls than boys on the context-free, domain-based, and negative affect, with PA being identical. The patterns of the loadings were consistent—with SWB loading more strongly onto the domain-based latent construct across all sub-groups. This was followed by strong positive loadings onto the context-free and PA latent construct. We found a moderate negative loading onto the NA across all subgroups. Future research should endeavor to ascertain the extent to which this model allows for cross-cultural comparisons. This may prove challenging. Previous research (see Casas and Rees, 2015; Casas, 2017) questioned the feasibility of cross-cultural comparisons of SWB measures given that measurement invariance was not tenable. This is not surprising as the adaptation and translation of measuring instruments, cultural response, and method bias limit the likelihood of achieving measurement invariance, which is requisite for meaningful cross-cultural comparisons. Regardless, given that in-country comparisons of children’s SWB are more significant than between-country comparisons (Rees, 2017) we recommend testing the model in individual country contexts. In the current study, we did not explore correlates of SWB. We recommend that future research explore correlates and predictors of children’s SWB using both the proposed model and the individual components as the outcome variable. Finally, given that we only tested a hierarchical structural model, we recommend further testing of other structural configurations of children’s SWB.

## Data Availability Statement

The raw data supporting the conclusions of this article are available from the International Society for Child Indicators upon reasonable request. Conditions and restrictions are applicable.

## Ethics Statement

Ethics procedures were aligned to country-specific requirements. Each of the 35 participating countries obtained ethics approval from the Institutional Review Boards of the Universities at which the researchers were based. Researchers obtained informed consent from the child participants; while parental informed consent was dependent on the prevailing practices and legal requirements in each country.

## Author Contributions

SS conceptualized the study with FC and SA, was the lead-author, wrote the introduction, wrote the method with SA, conducted the analysis with FC and SA, and wrote the discussion and conclusion with FC and SA. FC conceptualized the study with SS and SA, conducted the analysis with SS and SA, and wrote the discussion with SS and SA. SA conceptualized the study with SS and FC, wrote the method with SS, conducted part of the analysis with SS and FC, wrote the discussion with SS and FC, and assisted with manuscript editing. All authors contributed to the article and approved the submitted version.

## Conflict of Interest

The authors declare that the research was conducted in the absence of any commercial or financial relationships that could be construed as a potential conflict of interest.
