# Supplementary material for: The Structure of Children’s Subjective Well-being
Source: Front Psychol. 2021 Jun 11;12:650691. doi: 10.3389/fpsyg.2021.650691 (PMC8225927; doi:10.3389/fpsyg.2021.650691)
Supplement: Supplementary file 1 [file Table_1.docx]

Supplementary Table 1

*Intercepts: Configural model with unconstrained loadings and intercepts (Age and Gender)*

|  | **10-Years-Old** | | | **12-Years-Old** | | | **Boys** | | | **Girls** | | |
| --- | --- | --- | --- | --- | --- | --- | --- | --- | --- | --- | --- | --- |
|  | **Estimate** | **S.E.** | **C.R.** | **Estimate** | **S.E.** | **C.R.** | **Estimate** | **S.E.** | **C.R.** | **Estimate** | **S.E.** | **C.R.** |
| enjoylife | 8.916 | .009 | 972.745 | 8.598 | .010 | 872.540 | 8.811 | .010 | 918.595 | 8.726 | .010 | 902.357 |
| lifegoingwell | 8.800 | .009 | 936.496 | 8.460 | .010 | 834.818 | 8.670 | .010 | 878.037 | 8.620 | .010 | 870.963 |
| havegoodlife | 8.938 | .009 | 972.186 | 8.663 | .010 | 885.949 | 8.824 | .010 | 917.870 | 8.794 | .010 | 916.241 |
| thingslifeexcellent | 8.352 | .011 | 770.967 | 7.969 | .011 | 697.453 | 8.209 | .011 | 721.631 | 8.135 | .011 | 725.484 |
| happywithmylife | 9.013 | .009 | 985.627 | 8.668 | .010 | 846.228 | 8.893 | .010 | 916.544 | 8.817 | .010 | 891.412 |
| satisfiedpeoplelivewith | 9.011 | .009 | 1024.661 | 8.882 | .009 | 1016.545 | 8.895 | .009 | 966.393 | 9.011 | .009 | 1057.930 |
| satisfiedlifeasstudent | 8.599 | .010 | 889.298 | 8.297 | .010 | 826.201 | 8.340 | .011 | 792.806 | 8.596 | .009 | 918.154 |
| satisfiedfriends | 8.649 | .010 | 902.856 | 8.480 | .010 | 880.979 | 8.571 | .010 | 870.682 | 8.559 | .010 | 887.848 |
| satisfiedlocalarea | 8.604 | .010 | 841.434 | 8.233 | .011 | 766.479 | 8.430 | .011 | 779.327 | 8.450 | .010 | 816.836 |
| satisfiedthingshave | 9.056 | .008 | 1076.306 | 8.758 | .009 | 978.502 | 8.877 | .009 | 977.383 | 8.939 | .009 | 1042.867 |
| satisfiedtimeuse | 8.653 | .009 | 926.557 | 8.187 | .010 | 822.114 | 8.448 | .010 | 848.391 | 8.414 | .010 | 869.674 |
| satisfiedsafety | 8.923 | .009 | 1015.763 | 8.705 | .009 | 966.443 | 8.849 | .009 | 973.584 | 8.788 | .009 | 982.035 |
| satisfiedfreedom | 8.606 | .010 | 843.735 | 8.390 | .010 | 817.736 | 8.518 | .010 | 813.771 | 8.472 | .010 | 821.423 |
| satisfiedappearance | 8.506 | .011 | 790.298 | 8.078 | .012 | 693.580 | 8.411 | .011 | 763.113 | 8.213 | .012 | 706.404 |
| satisfiedlaterinlife | 8.501 | .011 | 799.134 | 8.270 | .011 | 784.637 | 8.439 | .011 | 781.593 | 8.342 | .011 | 780.281 |
| satisfiedhealth | 9.039 | .009 | 1049.048 | 8.815 | .009 | 969.568 | 8.924 | .009 | 979.931 | 8.944 | .009 | 1015.967 |
| feelinghappy | 8.806 | .009 | 947.331 | 8.444 | .010 | 845.326 | 8.669 | .010 | 888.522 | 8.616 | .010 | 882.027 |
| feelingcalm | 7.504 | .013 | 559.798 | 7.211 | .013 | 549.315 | 7.394 | .014 | 543.573 | 7.349 | .013 | 552.173 |
| feelingfullofenergy | 8.388 | .012 | 701.688 | 7.894 | .013 | 610.991 | 8.347 | .012 | 699.286 | 8.121 | .012 | 655.946 |
| feelingsad | 3.511 | .016 | 225.783 | 3.746 | .015 | 250.756 | 3.436 | .016 | 221.249 | 4.219 | .017 | 249.547 |
| feelingstressed | 4.089 | .017 | 239.809 | 4.203 | .016 | 254.919 | 4.060 | .017 | 236.759 | 4.425 | .017 | 268.097 |
| feelingbored | 4.149 | .017 | 248.827 | 4.537 | .016 | 281.076 | 4.277 | .017 | 253.601 | 8.596 | .009 | 918.154 |
