# Supplementary material for: The Structure of Children’s Subjective Well-being
Source: Front Psychol. 2021 Jun 11;12:650691. doi: 10.3389/fpsyg.2021.650691 (PMC8225927; doi:10.3389/fpsyg.2021.650691)
Supplement: Supplementary file 2 [file Table_2.docx]

Supplementary Table 2

*Intercepts: Metric model with constrained loadings (Age and Gender)*

|  | **10-Years-Old** | | | **12-Years-Old** | | | **Boys** | | | **Girls** | | |
| --- | --- | --- | --- | --- | --- | --- | --- | --- | --- | --- | --- | --- |
|  | **Estimate** | **S.E.** | **C.R.** | **Estimate** | **S.E.** | **C.R.** | **Estimate** | **S.E.** | **C.R.** | **Estimate** | **S.E.** | **C.R.** |
| enjoylife | 8.916 | .009 | 969.598 | 8.598 | .010 | 874.827 | 8.811 | .010 | 916.662 | 8.726 | .010 | 903.947 |
| lifegoingwell | 8.800 | .009 | 938.745 | 8.460 | .010 | 833.250 | 8.670 | .010 | 880.044 | 8.620 | .010 | 869.369 |
| havegoodlife | 8.938 | .009 | 980.983 | 8.663 | .010 | 879.746 | 8.824 | .010 | 921.235 | 8.794 | .010 | 913.580 |
| thingslifeexcellent | 8.352 | .011 | 775.158 | 7.969 | .011 | 694.125 | 8.209 | .011 | 721.595 | 8.135 | .011 | 725.514 |
| happywithmylife | 9.013 | .009 | 972.101 | 8.668 | .010 | 856.001 | 8.893 | .010 | 912.160 | 8.817 | .010 | 894.974 |
| satisfiedpeoplelivewith | 9.011 | .009 | 1025.729 | 8.882 | .009 | 1015.508 | 8.895 | .009 | 967.753 | 9.011 | .009 | 1056.583 |
| satisfiedlifeasstudent | 8.599 | .010 | 887.868 | 8.297 | .010 | 827.555 | 8.340 | .011 | 792.706 | 8.596 | .009 | 918.255 |
| satisfiedfriends | 8.649 | .009 | 912.978 | 8.480 | .010 | 870.736 | 8.571 | .010 | 874.617 | 8.559 | .010 | 883.971 |
| satisfiedlocalarea | 8.604 | .010 | 840.609 | 8.233 | .011 | 767.255 | 8.430 | .011 | 777.762 | 8.450 | .010 | 818.365 |
| satisfiedthingshave | 9.056 | .008 | 1076.460 | 8.758 | .009 | 978.357 | 8.877 | .009 | 986.573 | 8.939 | .009 | 1033.681 |
| satisfiedtimeuse | 8.653 | .009 | 921.955 | 8.187 | .010 | 826.298 | 8.448 | .010 | 848.247 | 8.414 | .010 | 869.813 |
| satisfiedsafety | 8.923 | .009 | 1022.351 | 8.705 | .009 | 960.200 | 8.849 | .009 | 974.417 | 8.788 | .009 | 981.227 |
| satisfiedfreedom | 8.606 | .010 | 849.748 | 8.390 | .010 | 812.042 | 8.518 | .010 | 812.168 | 8.472 | .010 | 822.963 |
| satisfiedappearance | 8.506 | .011 | 782.727 | 8.078 | .012 | 700.421 | 8.411 | .011 | 752.866 | 8.213 | .011 | 715.908 |
| satisfiedlaterinlife | 8.501 | .011 | 795.051 | 8.270 | .010 | 788.384 | 8.439 | .011 | 778.605 | 8.342 | .011 | 783.125 |
| satisfiedhealth | 9.039 | .009 | 1045.320 | 8.815 | .009 | 973.083 | 8.924 | .009 | 986.528 | 8.944 | .009 | 1009.407 |
| feelinghappy | 8.806 | .009 | 946.597 | 8.444 | .010 | 845.907 | 8.669 | .010 | 891.644 | 8.616 | .010 | 879.324 |
| feelingcalm | 7.504 | .013 | 564.364 | 7.211 | .013 | 544.863 | 7.394 | .014 | 543.411 | 7.349 | .013 | 552.326 |
| feelingfullofenergy | 8.388 | .012 | 697.324 | 7.894 | .013 | 614.820 | 8.347 | .012 | 694.909 | 8.121 | .012 | 659.828 |
| feelingsad | 3.511 | .016 | 225.088 | 3.746 | .015 | 251.588 | 3.436 | .016 | 220.556 | 3.823 | .015 | 249.151 |
| feelingstressed | 4.089 | .017 | 239.708 | 4.203 | .016 | 255.036 | 4.060 | .017 | 236.962 | 4.219 | .017 | 249.340 |
| feelingbored | 4.149 | .017 | 249.867 | 4.537 | .016 | 279.780 | 4.277 | .017 | 254.336 | 4.425 | .017 | 267.349 |
