# Supplementary material for: The Structure of Children’s Subjective Well-being
Source: Front Psychol. 2021 Jun 11;12:650691. doi: 10.3389/fpsyg.2021.650691 (PMC8225927; doi:10.3389/fpsyg.2021.650691)
Supplement: Supplementary file 3 [file Table_3.docx]

|  | **10-Years-Old** | | | **12-Years-Old** | | | **Boys** | | | **Girls** | | |
| --- | --- | --- | --- | --- | --- | --- | --- | --- | --- | --- | --- | --- |
|  | **Estimate** | **S.E.** | **C.R.** | **Estimate** | **S.E.** | **C.R.** | **Estimate** | **S.E.** | **C.R.** | **Estimate** | **S.E.** | **C.R.** |
| enjoylife | 8.911 | .008 | 1061.457 | 8.603 | .009 | 929.581 | 8.796 | .009 | 966.418 | 8.739 | .009 | 998.096 |
| lifegoingwell | 8.797 | .009 | 1000.137 | 8.463 | .010 | 867.635 | 8.676 | .009 | 906.838 | 8.615 | .009 | 937.206 |
| havegoodlife | 8.965 | .009 | 1050.119 | 8.640 | .009 | 913.276 | 8.840 | .009 | 950.636 | 8.780 | .009 | 981.708 |
| thingslifeexcellent | 8.328 | .010 | 868.714 | 7.991 | .011 | 760.751 | 8.203 | .010 | 792.099 | 8.141 | .010 | 816.073 |
| happywithmylife | 9.003 | .009 | 1041.080 | 8.603 | .009 | 929.581 | 8.884 | .009 | 946.085 | 8.825 | .009 | 978.645 |
| satisfiedpeoplelivewith | 9.065 | .007 | 1262.147 | 8.832 | .007 | 1194.528 | 8.961 | .007 | 1227.093 | 8.957 | .007 | 1210.567 |
| satisfiedlifeasstudent | 8.575 | .008 | 1070.398 | 8.322 | .008 | 1005.688 | 8.491 | .008 | 1104.064 | 8.563 | .008 | 1032.109 |
| satisfiedfriends | 8.672 | .008 | 1143.955 | 8.457 | .008 | 1086.871 | 8.567 | .009 | 981.806 | 8.439 | .008 | 1098.118 |
| satisfiedlocalarea | 8.551 | .008 | 1012.107 | 8.291 | .009 | 950.410 | 8.443 | .007 | 1190.327 | 8.908 | .009 | 972.532 |
| satisfiedthingshave | 9.034 | .007 | 1242.616 | 8.782 | .008 | 1158.125 | 8.912 | .008 | 998.496 | 8.428 | .008 | 1176.635 |
| satisfiedtimeuse | 8.571 | .008 | 1043.541 | 8.277 | .009 | 965.066 | 8.433 | .008 | 1102.147 | 8.816 | .009 | 987.761 |
| satisfiedsafety | 8.959 | .008 | 1159.059 | 8.669 | .008 | 1073.260 | 8.820 | .009 | 941.027 | 8.492 | .008 | 1091.547 |
| satisfiedfreedom | 8.658 | .009 | 988.995 | 8.340 | .009 | 916.731 | 8.497 | .010 | 859.727 | 8.314 | .009 | 932.025 |
| satisfiedappearance | 8.459 | .009 | 898.532 | 8.130 | .010 | 827.887 | 8.320 | .009 | 921.816 | 8.388 | .010 | 857.442 |
| satisfiedlaterinlife | 8.540 | .009 | 961.932 | 8.236 | .009 | 900.294 | 8.392 | .008 | 1049.547 | 8.487 | .009 | 914.152 |
| satisfiedhealth | 9.053 | .007 | 1226.788 | 8.800 | .008 | 1147.050 | 8.936 | .008 | 1180.649 | 8.932 | .008 | 1170.273 |
| feelinghappy | 8.824 | .009 | 975.370 | 8.426 | .010 | 863.711 | 8.688 | .009 | 899.330 | 8.598 | .009 | 920.516 |
| feelingcalm | 7.515 | .011 | 711.831 | 7.201 | .011 | 660.736 | 7.408 | .011 | 675.120 | 7.337 | .011 | 686.298 |
| feelingfullofenergy | 8.308 | .010 | 826.155 | 7.982 | .011 | 753.252 | 8.272 | .010 | 800.319 | 8.198 | .010 | 822.384 |
| feelingsad | 3.505 | .014 | 243.451 | 3.752 | .014 | 272.149 | 3.502 | .014 | 264.341 | 3.759 | .014 | 243.184 |
| feelingstressed | 4.022 | .015 | 265.421 | 4.267 | .015 | 291.533 | 4.012 | .015 | 283.634 | 4.266 | .015 | 263.999 |
| feelingbored | 4.227 | .015 | 288.952 | 4.459 | .014 | 314.025 | 4.229 | .015 | 306.696 | 4.472 | .015 | 286.911 |

Supplementary Table 3

*Intercepts: Scalar model with constrained loadings and intercepts (Age and Gender)*
