# Supplementary material for: The Structure of Children’s Subjective Well-being
Source: Front Psychol. 2021 Jun 11;12:650691. doi: 10.3389/fpsyg.2021.650691 (PMC8225927; doi:10.3389/fpsyg.2021.650691)
Supplement: Supplementary file 4 [file Table_4.docx]

Supplementary Table 4

*Factor loadings, intercepts, and variances from the final model (Scalar) with constrained loadings and intercepts (Age and Gender)*

|  | **10-Years-Old** | | **12-Years-Old** | | **Boys** | | **Girls** | |
| --- | --- | --- | --- | --- | --- | --- | --- | --- |
|  | **Factor loadings*** | **Intercepts** | **Factor loadings** | **Intercepts** | **Factor loadings** | **Intercepts** | **Factor loadings** | **Intercepts** |
| enjoylife | 1.000 (.786) | 8.911 | 1.000 (.839) | 8.603 | 1.000 (.799) | 8.796 | 1.000 (.826) | 8.739 |
| lifegoingwell | 1.085 (.837) | 8.797 | 1.085 (.882) | 8.463 | 1.084 (.846) | 8.676 | 1.084 (.873) | 8.615 |
| havegoodlife | 1.053 (.837) | 8.965 | 1.053 (.884) | 8.640 | 1.056 (.848) | 8.840 | 1.056 (.876) | 8.780 |
| thingslifeexcellent | 1.090 (.732) | 8.328 | 1.090 (.783) | 7.991 | 1.091 (.738) | 8.203 | 1.091 (.776) | 8.141 |
| happywithmylife | 1.052 (.820) | 9.003 | 1.052 (.858) | 8.603 | 1.049 (.827) | 8.884 | 1.049 (.850) | 8.825 |
| satisfiedpeoplelivewith | .917 (.555) | 9.065 | .917 (.596) | 8.832 | .921 (.561) | 8.961 | .921 (.595) | 8.957 |
| satisfiedlifeasstudent | 1.000 (.546) | 8.575 | 1.000 (.566) | 8.322 | 1.000 (.528) | 8.491 | 1.000 (.588) | 8.563 |
| satisfiedfriends | .850 (.476) | 8.672 | .850 (.496) | 8.457 | .861 (.492) | 8.567 | .861 (.491) | 8.439 |
| satisfiedlocalarea | 1.025 (.530) | 8.551 | 1.025 (.541) | 8.291 | 1.031 (.532) | 8.443 | 1.031 (.550) | 8.908 |
| satisfiedthingshave | .997 (.630) | 9.034 | .997 (.632) | 8.782 | 1.005 (.626) | 8.912 | 1.005 (.641) | 8.428 |
| satisfiedtimeuse | 1.162 (.657) | 8.571 | 1.162 (.663) | 8.277 | 1.165(.656) | 8.433 | 1.165 (.664) | 8.816 |
| satisfiedsafety | 1.148 (.699) | 8.959 | 1.148 (.720) | 8.669 | 1.166 (.718) | 8.820 | 1.166 (.718) | 8.492 |
| satisfiedfreedom | 1.255 (.659) | 8.658 | 1.255 (.691) | 8.340 | 1.267 (.677) | 8.497 | 1.267 (.678) | 8.314 |
| satisfiedappearance | 1.300 (.634) | 8.459 | 1.300 (.639) | 8.130 | 1.299 (.650) | 8.320 | 1.299 (.623) | 8.388 |
| satisfiedlaterinlife | 1.204 (.598) | 8.540 | 1.204 (.653) | 8.236 | 1.209 (.624) | 8.392 | 1.209 (.625) | 8.487 |
| satisfiedhealth | .998 (.613) | 9.053 | .998 (.626) | 8.800 | 1.002 (.619) | 8.936 | 1.002 (.624) | 8.932 |
| feelinghappy | 1.000 (.771) | 8.824 | 1.000 (.810) | 8.426 | 1.000 (.778) | 8.688 | 1.000 (.800) | 8.598 |
| feelingcalm | .788 (.426) | 7.515 | .788 (.481) | 7.201 | .793 (.442) | 7.408 | .793 (.468) | 7.337 |
| feelingfullofenergy | .818 (.487) | 8.308 | .818 (.512) | 7.982 | .824 (.519) | 8.272 | .824 (.524) | 8.198 |
| feelingsad | 1.000 (.703) | 3.505 | 1.000 (.692) | 3.752 | 1.000 (.699) | 3.502 | 1.000 (.698) | 3.759 |
| feelingstressed | .993 (.636) | 4.022 | .993 (.620) | 4.267 | .991 (.628) | 4.012 | .991 (.627) | 4.266 |
| feelingbored | .939 (.618) | 4.227 | .939 (.596) | 4.459 | .943 (.611) | 4.229 | .943 (.611) | 4.472 |
|  |  |  |  |  |  |  |  |  |
| **Variances** | |  |  |  |  |  |  |  |
| Cognitive Context-Free Life Satisfaction | | 0.645 |  | 0.723 |  | 0.660 |  | 0.707 |
| Cognitive Domain-Based Life Satisfaction | | 0.364 |  | 0.389 |  | 0.374 |  | 0.385 |
| Positive Affect | | 0.092 |  | 0.105 |  | 0.097 |  | 0.103 |
| Negative Affect | | 0.116 |  | 0.111 |  | 0.114 |  | 0.114 |

* Standardised loadings in parenthesis

All values significant at p < .001
